# The Association of Dyslipidemia and Atherogenic Indices With Glycemic Control in Diabetic Dyslipidemia Patients: A Real-World Landscape

**DOI:** 10.7759/cureus.45985

**Published:** 2023-09-26

**Authors:** Isaac J Bage, Sadishkumar Kamalanathan, Sandhiya Selvarajan, Jayaprakash Sahoo, Mathaiyan Jayanthi, Dukhabandhu Naik

**Affiliations:** 1 Endocrinology, Jawaharlal Institute of Postgraduate Medical Education and Research, Puducherry, IND; 2 Clinical Pharmacology, Jawaharlal Institute of Postgraduate Medical Education and Research, Puducherry, IND; 3 Pharmacology, Jawaharlal Institute of Postgraduate Medical Education and Research, Puducherry, IND

**Keywords:** diabetic dyslipidemia, pattern of dyslipidemia, hba1c, glycemic control, atherogenic indices

## Abstract

Background: Dyslipidemia is an important comorbid factor of type 2 diabetes mellitus (T2DM) that increases the risk of cardiovascular diseases. This study aimed to assess the pattern of dyslipidemia and atherogenic indices and determine its relation with glycemic control.

Methods:A cross-sectional study enrolled 382 patients with diabetic dyslipidemia. The socio-demographics data, clinical features, and laboratory parameters were collected. The baseline lipid parameters such as total cholesterol (TC), triglyceride (TG), low-density lipoprotein cholesterol (LDL-C), high-density lipoprotein cholesterol (HDL-C), and glycated hemoglobin (HbA1C) were measured. Atherogenic indices such as TC/HDL-C ratio, TG/HDL-C ratio, LDL-C/HDL-C ratio, non-HDL-C/HDL-C and atherogenic index of plasma (AIP) [log10 (TG/HDL-C)] were calculated. T2DM patients were classified into three groups based on the degree of glycemic control: Good glycemic control (HbA1C<7%), fair control (HbA1C 7-8%), and poor control (HbA1C>8%).

Results:The population’s mean age was 48.60±6.15 years, with 145 (38%) males. We found mixed dyslipidemia as the most prevalent (36.1%) form of dyslipidemia in our patients. The most common pattern in atherogenic indices was AIP (94.2%). HbA1c was positively correlated with duration of diabetes (r*=*0.253, p<0.001). In multivariate logistic regression analysis, duration of diabetes (>10 years) was significantly associated with poor glycemic control with an odds ratio (OR) of 2.31(95% CI; 1.25-4.24, p=0.007).

Conclusion:The present study indicated that neither the pattern of dyslipidemia nor the atherogenic indices were markers of poor glycemic control among South Indian patients attending our tertiary care institute. However, duration of diabetes was significantly associated with poor glycemic control.

## Introduction

Type 2 diabetes mellitus (T2DM) is one of the leading causes of mortality and morbidity worldwide. It is a major global public health problem associated with a two to four-fold higher risk of cardiovascular disease (CVD) [1]. The International Diabetes Federation (IDF) estimated that the worldwide burden of diabetes has tripled from 151 million in 2000 to 537.5 million of the global population today. Its prevalence in the world, Southeast Asia, and India was 10.5%, 8.8%, and 9.6%, respectively in 2021; and will rise to 12.5%, 11.5%, and 10.9% by 2045 [2].

Glycated hemoglobin (HbA1c), a marker of glycemic control, is considered a better predictor of coronary artery disease (CAD) than fasting or two-hour plasma glucose in patients with diabetes [3]. HbA1C reflects the average blood glucose level in T2DM patients over three months. Achieving the HbA1C target value of less than 7% has been shown to reduce diabetic vascular complications in diabetic patients [4].

Cardiovascular diseases (CVD), encompassing CAD, myocardial infarction (MI), congestive heart failure, and stroke is the leading cause of death in patients with T2DM, and dyslipidemia is believed to be an important factor in its pathogenesis. The primary component of diabetic dyslipidemia contributing to the risk of CVD has been elevated low-density lipoprotein cholesterol (LDL-C). Additionally, LDL-C is transformed into small dense LDL-C, which is more atherogenic [5]. Approximately 80% of the deaths occurring in patients with type 2 diabetes are accounted for by cardiovascular disease (CVD), and Asian Indians have a higher risk than Caucasians. Patients with T2DM have an increased prevalence of dyslipidemia, such as increased total cholesterol (TC), increased triglyceride (TG), high LDL-C, and reduced HDL-C [6]. In patients with T2DM with dyslipidemia, the risk of developing myocardial infarction or stroke is increased two to threefold, and the risk of death is increased twofold, independent of other known risk factors for cardiovascular disease in T2DM patients with dyslipidemia. Early treatment to normalize the circulating lipid has been shown to reduce cardiovascular disease in diabetic patients [7].

Recently, various indices like Castelli's risk index-I (CRI-I), Castelli's risk index-II (CRI- II), atherogenic coefficient (AC), and Atherogenic Index of Plasma (AIP) have emerged as potential markers to evaluate cardiovascular risk in T2DM patients. The Castelli Risk Index (CRI) is calculated from TC, LDL-C, and HDL-C and is divided into CRI-I and CRI-II, respectively. CRI-I is calculated from the formula: CRI-I=TC/HDL-C, CRI-II is calculated from the formula: CRI-II=LDL-C/HDL-C, and AC from the formula: AC=(TC-HDL-C)/HDL-C. Both CRI and AC are independent risk factors associated with CAD. AIP calculated through the formula: AIP=log10 (TG/HDL-C) is a good tool for determining the fractionated esterification rate of HDL-C and is more valuable than the routine lipid profile. It can also be used as a diagnostic marker for cardiovascular (CV) events when other atherogenic parameters appear normal [8]. Presently, there is a dearth of meaningful analysis between diabetes and dyslipidemia indices in real-world scenarios.

Very few studies are available on the relationship between atherogenic indices, patterns of dyslipidemia, and glycemic control in South India. Therefore, this study was conducted to explore the pattern of dyslipidemia, atherogenic indices, and their association with glycemic control in diabetic dyslipidemia patients.

## Materials and methods

The study was conducted in the Endocrinology and Diabetic clinics of a tertiary care institute in South India after approval by the lnstitutional Ethical Committee of Jawaharlal Institute of Postgraduate Medical Education and Research (JIPMER), Puducherry, India (JEC Reg.No:JIP/IEC/2017/0123). The study period was from July 2017 to October 2021. Written informed consent was obtained from all patients prior to the commencement of the study. Patients' socio-demographic data, including age, gender, smoking history, alcohol consumption, family history of diabetes, height, weight, body mass index (BMI), and blood pressure (BP), were collected. T2DM patients with diabetic dyslipidemia aged 30-60 years were screened for inclusion in the study; those with either HbA1C >10% or chronic kidney disease (CKD) with estimated glomerular filtration rate<60 ml/min/1.73m^2^, abnormal liver function test (LFT) (more than three times ULN (upper limit of normal) range) or heart failure NYHA (New York Heart Association) Class III or more were excluded from the study. Venous blood samples of 3 ml were collected from all the patients after at least eight hours of fasting using a disposable syringe under sterile conditions in a clot activator tube. Blood samples were centrifuged at 3000 rpm for 10 minutes and the serum separated. The lipid parameters were measured using a fully automated clinical chemistry analyzer (AU680, Beckman Coulter). LDL-C level was estimated directly rather than calculating using the Friedewald equation. Additionally, 2 ml of blood samples were collected for glycated hemoglobin (HbA1c) in potassium Ethylenediaminetetraacetic Acid (K2EDTA) vacutainer tubes. HbA1c was measured in HbA1c high-performance liquid chromatography (HPLC) analyzer Bio-Rad D 10 (Bio-Rad Laboratories, Inc., CA, USA)

Variable categorization and definition

BMI was calculated using the formula BMI (kg/m^2^)=(weight in kilograms)/height in meters)^2^ was classified as normal (18.5-22.9 kg/m^2^), overweight (23-24.9 kg/m^2^), obese (≥25 kg/m^2^). Blood pressure (BP) was categorized as normal (<120/80 mmHg), pre-hypertension (120/80-139/89 mmHg), and hypertension (≥140/90 mmHg). Alcohol consumption and smoking were classified as 'yes'/'no' on documentation of their previous or present drinking or smoking habits. Patients were classified into three groups based on their HbA1c levels. Group1: subjects with good glycemic control (HbA1c<7%); Group2: subjects with fair glycemic control (HbA1c 7-8%); Group3: subjects with poor glycemic control (HbA1c>8%) [9]. T2DM, for study purposes, was defined as patients taking regular antidiabetic drugs to maintain blood glucose levels in a normal range, while dyslipidemia was having a variable combination of total cholesterol (TC ≥200mg/dl), triglyceride (TG ≥150mg/dl), low-density lipoprotein-cholesterol (LDL-C ≥100mg/dl) and high-density lipoprotein-cholesterol (HDL-C<40mg/dl for men/ HDL-C<50mg/dl for women). In addition, a CVD risk assessment was done and those reaching the targets of LDL-C<70mg/dl and LDL-C<55mg/dl were assessed. Abnormality of lipid indices was defined as non-HDL-C ≥130mg/dl, Atherogenic Coefficient (AC) >3.5, Castelli's risk index-II (CRI- II) >2.5, TG/ HDL-C >3.5, Castelli's Risk Index-I (CRI-I) >4 and Atherogenic Index of plasma (AIP) >0.24 [10,11]. The calculated sample size was 384 patients using the formula N=Z2*P(1-P)/d2 at a 95% confidential interval and a 5% margin of error.

Statistical analysis

Statistical analysis was done in SPSS software, version 21.0 (IBM Corp., Armonk, NY). Descriptive statistics were used to summarize continuous and categorical data. Continuous data were presented as mean ± standard deviation (SD) and compared using one-way analysis of variance (ANOVA) with Bonferroni post hoc test. The Kolmogorov-Smirnov test was used to check for the normality of continuous data. Categorical data were described as percentages and frequencies and were compared using the Chi-square test. The correlation between HbA1C and lipid profile, atherogenic indices, and other variables was calculated using Pearson's or Spearman's rank correlation test. Multivariate logistic regression analysis explored the association between HbA1C, anthropometric, lipid parameters, and atherogenic indices. The odds ratio (OR) and 95% confidence intervals (95%CI) were calculated. A p<0.05 in the 2-sided test was considered statistically significant.

## Results

Characteristics of the study participants

Three hundred eighty-two diabetic dyslipidemia patients, including 38% (n=145) male, were recruited in this study. The mean age of the patients was 48.60±6.15 (years). Among them, 27.5% (n=105) had hypertension, 8.1% (n=31) were smokers, and 4.2% (n=16) were alcoholics. A family history of diabetes was present in 89% (n=341) of cases. The mean BMI was 27.40 kg/m^2^, and 70.2% (n=268) were obese. The average duration of diabetes was 6.9 years (Table 1).

**Table 1:** Characteristics of the study participants (n=382) HTN, hypertension; BMI, body mass index; BP, blood pressure; HbA1C, Hemoglobin A1C; LDL-C, low-density lipoprotein; HDL-C, high-density lipoprotein. The parameters are represented as mean ± SD or n (%).

Variable	All Statistic (mean ± SD or n (%))
Age (years)	48.60±6.15
Age category (30-40: 41-50: 51-60 years)	(12: 43: 45)
Gender (Male)	145(38)
Family history of diabetes	341(89)
Duration of diabetes (years)	6.9±5.27
Duration of diabetes (years) category (≤7: 7-10: >10 years)	(58: 24: 18)
BMI kg/m^2^	27.40±4.86
Body mass index status (normal: overweight: obese)	(18: 12: 70)
Systolic BP (mmHg)	130.65±17.57
Diastolic BP (mmHg)	81.02±10.42
HTN category (normal: pre HTN: HTN)	(43: 30: 27)
Smoker	16(4)
Alcoholism	31(8)
HbA1C	8.01±0.89(%)
Degree of glycemic control (good: fair: poor control)	(9: 34: 57)
Total cholesterol (mg/dl)	202.46±45.26
High total cholesterol	194(51)
Triglyceride (mg/dl)	189.72±86.86
High triglyceride	237(62)
LDL-C (mg/dl)	125.88±40.3
High LDL-C (≥100 mg/dl)	285(75)
High LDL-C (≥70 mg/dl)	358(93.7)
High LDL-C (≥55 mg/dl)	372(97.4)
HDL-C (mg/dl)	43.96±10.90
Low HDL-C	286(75)
Non-HDL-C (mg/dl)	158.11±43.87
High Non-HDL-C	276(72.3)

Regarding glycemic control, 57.3% (n=219) of the patients had poor glycemic control, 34% (n=130) had fair control, and 9% (n=33) had good control., The mean duration of diabetes was significantly higher in poor glycemic control than in good glycemic control (8.06±5.69 vs 4.48±4.36, p<0.001), as shown in Table 2. 

**Table 2:** Characteristics of the study participants categorized by HbA1C HTN, hypertension; BMI, body mass index; BP, blood pressure; HbA1C, Hemoglobin A1C; LDL-C, low-density lipoprotein; HDL-C, high-density lipoprotein. A p-value <0.05 was considered as significant.

Variable	Statistic (mean ± SD or n(%))	Good control (mean ± SD/ (%), n= 33)	Fair control (mean ± SD/ (%), n=130)	Poor control (mean ± SD/ (%), n=219)	p-value
Age (years)	48.60±6.15	48.57±6.21	49.54±5.75	48.69±6.41	0.64
Age category (years)					
30-40	47(12.3)	5(15.2)	12(9.2)	30(13.7)	0.74
41-50	164(42.9)	14(42.4)	56(43.1)	94(42.9)	
51-60	171(44.8)	14(42.4)	62(47.7)	95(43.4)	
Gender (Male)	145(38)	14(42.4)	50(38.5)	84(38.4)	0.90
Family history of diabetes	341(89.3)	32(96.9)	116(89.2)	193(88.1)	0.31
Duration of diabetes (years)	6.9±5.27	4.48±4.36	5.77±4.58	8.06±5.69	<0.001
Duration of diabetes category (years)					
≤7	223(58.4)	26(78.1)	87(66.9)	110(50.2)	0.002
7-10	92(24.1)	4(12.1)	28(21.5)	60(27.4)	
>10	67(17.5)	3(9.1)	15(11.5)	49(22.4)	
Weight (kg)	65.60±12.30	66.45±11.25	66.99±12.37	64.58±12.32	0.23
BMI kg/m^2^	27.40±4.86	27.68±4.30	27.85±4.83	27.05±5.05	0.19
Body weight status					
Overweight	47(12.3)	4(12.1)	17(13.1)	26(11.9)	0.65
Obese	268(70.2)	25(75.8)	94(72.3)	149(68)	
Systolic BP (mmHg)	130.65±17.57	130.84±19.77	131.63±16.31	130.98±18.13	0.94
Diastolic BP (mmHg)	81.02±10.42	83.51±13.67	79.86±11.75	81.68±8.86	0.12
HTN	105(27.5)	7(21.2)	34(26.2)	64(29.2)	0.68
Smoker	16(4.2)	0	8(6.2)	8(3.7)	0.24
Alcoholism	31(8.1)	2(6.1)	12(9.2)	17(7.8)	0.81
Total cholesterol (mg/dl)	202.46±45.26	202.87±33.59	198.20±50.53	204.07±45.17	0.51
High total cholesterol	194(50.8)	18(54.5)	58(44.6)	118(53.9)	0.22
Triglyceride (mg/dl)	189.72±86.86	193.18±79.64	177.40±81.29	195.18±89.33	0.16
High triglyceride	237(62)	24(72.7)	73(56.2)	140(63.9)	0.14
LDL-C (mg/dl)	125.88±40.3	117.57±29.88	123.59±43.51	127.94±39.55	0.31
High LDL-C (≥100 mg/dl)	285(74.6)	25(75.8)	90(69.2)	170(77.6)	0.21
High LDL-C (≥70 mg/dl)	358(93.7)	32(97)	119(91.5)	207(94.5)	0.39
High LDL-C (≥55 mg/dl)	372(97.4)	32(97)	127(97.7)	213(97.3)	0.95
HDL-C (mg/dl)	43.96±10.90	44.66±12.90	44.41±11.78	43.40±9.82	0.63
Low HDL-C	286(74.9)	24(72.7)	92(70.8)	170(77.6)	0.34
Non-HDL-C (mg/dl)	158.11±43.87	158.21±31.44	153.78±48.03	160.67±42.83	0.36
High Non-HDL-C	276(72.3)	24(73)	83(63.8)	166(75.8)	0.05

Dyslipidemia patterns

Table 3 shows the distribution of dyslipidemia patterns based on glycemic control. 

**Table 3:** Pattern of dyslipidemia and atherogenic indices categorized by HbA1C TC, total cholesterol; TG, triglyceride; LDL-C, low-density lipoprotein; HDL-C, high-density lipoprotein; AIP, Atherogenic index of plasma. A p-value <0.05 was considered as significant.

Variable	Statistic (mean ± SD or n(%))	Good control (mean ± SD/ (%), n= 33)	Fair control (mean ± SD/ (%), n=130)	Poor control (mean ± SD/ (%), n=219)	p-value
TC/HDL-C ratio	4.77±1.28	4.82±1.28	4.64±1.34	4.85±1.24	0.32
High TC/HDL-C ratio	273(71.5)	24(72.7)	85(65.4)	164(74.9)	0.16
TG/HDL-C ratio	4.62±2.52	4.69±2.31	4.31±2.30	4.79±2.66	0.22
High TG/HDL-C ratio	234(61.3)	21(63.6)	76(58.5)	137(62.6)	0.71
LDL/HDL-C ratio	2.97±1.08	2.82±1.02	2.89±1.11	3.05±1.06	0.28
High LDL/HDL-C ratio	155(42)	12(36.4)	42(36.2)	97(46.4)	0.14
Non-HDL-C/HDL-C ratio	3.77±1.28	3.82±1.28	3.64±1.34	3.85±1.24	0.32
High non-HDL-C/HDL-C ratio	264(70.8)	23(71.9)	82(64.6)	159(74.3)	0.16
Atherogenic Index of plasma (AIP)	0.60±0.23	0.61±0.023	0.57±0.23	0.62±0.22	0.18
High atherogenic Index of plasma (AIP) status	360(94.2)	31(93.9)	120(92.3)	209(95.4)	0.48
Isolated dyslipidemia					
High TG, n(%)	10(2.6)	2(6.1)	2(1.5)	6(2.7)	0.34
High LDL-C	40(10.5)	2(6.1)	18(13.8)	20(9.1)	0.26
Low HDL-C	32(8.4)	2(6.1)	14(10.8)	16(7.3)	0.46
Combined dyslipidemia					
High TG and high LDL-C	40(10.5)	4(12.1)	14(10.8)	22(10)	0.92
High TG and low HDL-C	49(12.8)	3(9.1)	20(15.4)	26(11.9)	0.51
High LDL-C and low HDL-C	67(17.5)	4(12.1)	21(16.2)	42(19.2)	0.53
Mixed dyslipidemia					
High TG, high LDL-C and low HDL-C	138(36.1)	15(45.5)	37(28.5)	86(39.3)	0.06

High TC was seen in 51% (n=194), high TG in 62% (n=237), high LDL-C, i.e.≥100 mg/dl in 75% (n=285), low HDL-C in 76% (n=286), and high non-HDL-C in 72% (n=276), respectively. Similarly, the prevalence of low HDL-C was 68.8% (n=163) in females and 51% (n=74) in males, respectively.

Among patients with isolated dyslipidemia, high LDL-C was seen in 10.5% (n=40), low HDL-C in 8.4% (n=32), and high TG in 2.6% (n=10), respectively. Among subjects with combined dyslipidemia, the prevalence of high LDL-C and low HDL-C was 17.5% (n=67), while that of a combination of high TG and low HDL-C was 12.8% (n=49). The prevalence of a combined high TG and high LDL-C was 10.5% (n=40). The prevalence of combined mixed dyslipidemia of high TG, high LDL-C, and low HDL-C was 36.1% (n=138). The prevalence of high TC/HDL-C ratio was 71.5% (n=273), while that of high TG/HDL-C ratio, high LDL-C/HDL-C ratio, non-HDL-C/HDL-C ratio, and AIP were 61.3% (n=234), 42% (n=155), 70.8% (n=264) and 94.2% (n=360) respectively. All the diabetic dyslipidemia patients were taking statins. Our study showed that 97 (25.3%), 24 (6.2%), and 10 (2.6%) patients achieved the LDL-C targets of <100, <70, and <55 mg/dl, respectively. Similarly, the target of HDL-C (>50 mg/dl) in females was achieved in 74 (31.2%) patients, while the HDL-C (>40mg/dl) in males was achieved in 71 (49%). The targets achieved of triglyceride (TG <150 mg/dl) in 145 (38%) as per American Diabetes Association (ADA), 2023 guidelines [12]. Further, the patients within the target range of HbA1C<6.5% and <7% were 11 (2.9%) and 33 (8.7%) respectively.

Correlation analysis of HbA1C with lipids parameter and other variables

The correlation analyses were performed to investigate the correlation between HbA1C and other continuous variables such as age, BMI, duration of diabetes, lipid parameter, atherogenic indices, isolated, combined, and mixed dyslipidemia, as summarized in Table 4.

**Table 4:** Correlation between HbA1C and other variables TG, triglyceride; LDL-C, low-density lipoprotein; HDL-C, high-density lipoprotein; BMI, body mass index; AIP, Atherogenic index of plasma. A p-value <0.05 was considered as significant.

Variable	Correlation	p-value
Age(years)	-0.015	0.76
Duration of diabetes(years)	0.253	<0.001
BMI (kg/m^2^)	-0.061	0.23
Total cholesterol (mg/dl)	0.008	0.88
Triglyceride (mg/dl)	0.088	0.08
LDL-C (mg/dl)	0.013	0.81
HDL-C (mg/dl)	-.074	0.15
Non-HDL-C (mg/dl)	0.026	0.61
TC/HDL-C ratio	0.042	0.41
TG/HDL-C ratio	0.092	0.07
LDL/HDL-C ratio	0.038	0.45
non-HDL-C/HDL-C ratio	0.042	0.41
Atherogenic Index of Plasma (AIP)	0.071	0.16
Isolated dyslipidemia		
High TG	0.055	0.28
High LDL-C	0.018	0.72
Low HDL-C	0.064	0.21
Combined dyslipidemia		
High TG and high LDL-C	0.012	0.99
High TG and low HDL-C	0.078	0.12
High LDL-C and low HDL-C	0.058	0.22
Mixed dyslipidemia		
High LDL-C, high TG and low HDL-C	0.077	0.13

HbA1c was positively correlated with duration of diabetes (r=0.253, p<0.001). In multivariate logistic regression analysis, the duration of diabetes (>10 years) was significantly associated with worse glycemic control with an OR of 2.31(95% CI; 1.25-4.24, p=0.007) as shown in Table 5.

**Table 5:** Multivariate logistic regression showing the relationship between lipid fractions and other variables with glycemic status TG, triglyceride; LDL-C, low-density lipoprotein; HDL-C, high-density lipoprotein; HTN, hypertension; AIP, Atherogenic index of plasma. Values are presented as OR (95% CI), OR, odds ratio, CI, Confidence Interval. ‘REF’ is represented as the reference.

Variable	Good control		Fair control (mean ± SD (%), n=169)		Poor control (mean ± SD (%), n=237)	
	OR (95% CI)	p-value	OR (95% CI)	p-value	OR (95% CI)	p-value
Age (>50 years)	REF	REF	0.90(0.39-2.05)	0.81	0.98(0.44-2.18)	0.97
Duration of diabetes (>10 years)	REF	REF	1.42(0.75-2.68)	0.27	2.31(1.25-4.24)	0.007
Obesity	REF	REF	0.89(0.51-1.53)	0.67	0.75(0.45-1.26)	0.29
HTN	REF	REF	1.09(0.68-1.74)	0.71	1.10(0.70-1.72)	0.66
High total cholesterol (mg/dl)	REF	REF	0.67(0.31-1.44)	0.31	0.97(0.46-2.03)	0.94
High triglyceride (mg/dl)	REF	REF	0.48(0.20-1.11)	0.08	0.66(0.29-1.50)	0.32
LDL-C (≥100 mg/dl)	REF	REF	0.72(0.29-1.73)	0.46	1.11(0.47-2.61)	0.81
LDL-C (≥70 mg/dl)	REF	REF	0.33(0.04-2.71)	0.31	0.53(0.06-4.28)	0.55
LDL-C (≥55 mg/dl)	REF	REF	1.32(0.13-13.11)	0.81	1.10(0.12-9.51)	0.92
Low HDL-C (mg/dl)	REF	REF	0.90(0.38-2.12)	0.82	1.30(0.56-2.98)	0.53
High Non-HDL-C (mg/dl)	REF	REF	0.39(0.15-1.01)	0.06	0.69(0.27-1.17)	0.44
High TC/HDL-C ratio	REF	REF	0.70(0.30-1.65)	0.42	1.11(0.49-2.55)	0.79
High TG/HDL-C ratio	REF	REF	0.80(0.36-1.77)	0.58	0.95(0.44-2.05)	0.91
High LDL/HDL-C ratio	REF	REF	0.99(0.44-2.24)	0.99	1.51(0.79-3.24)	0.28
High non HDL-C/HDL-C ratio	REF	REF	0.71(0.30-1.67)	0.43	1.13(0.49-2.59)	0.77
High atherogenic index of plasma (AIP)	REF	REF	1.03(0.20-5.11)	0.93	1.53(0.30-7.65)	0.59
Isolated dyslipidemia						
High TG	REF	REF	0.39(0.051-3.11)	0.36	0.55(0.10-2.98)	0.4
High LDL-C	REF	REF	3.51(0.73-16.8)	0.11	1.89(0.39-8.65)	0.44
Low HDL-C	REF	REF	2.73(0.55-13.9)	0.21	1.47(0.30-7.01)	0.62
Combined dyslipidemia						
High TG and high LDL-C	REF	REF	1.36(0.39-4.97)	0.62	1.01(0.30-3.39)	0.98
High TG and low HDL-C	REF	REF	2.61(0.67-9.97)	0.16	1.59(0.20-5.11)	0.48
High LDL-C and low HDL-C	REF	REF	2.04(0.60-6.90)	0.24	1.93(0.60-6.11)	0.26
Mixed dyslipidemia	REF	REF	0.47(0.21-1.06)	0.07	0.74(0.35-1.58)	0.44

## Discussion

Our study aimed to determine the pattern of dyslipidemia and atherogenic indices in diabetic dyslipidemia patients and to explore their association with glycemic control. Mixed dyslipidemia (high TG, high LDL-C, and low HDL-C) was the most common abnormality in diabetic dyslipidemia patients. When comparing our results with other studies, the prevalence of mixed dyslipidemia was higher than the previous studies in type 2 diabetes, with dyslipidemia reported among the USA population (21%) [13], and Canada (3.2%) [14]. However, mixed dyslipidemia was found to be 38.7% among the French population, similar to our study [15]. Our finding showed that combined dyslipidemia (high LDL-C and low HDL-C) was the second most common in the study population. Parik et al. found that the prevalence of combined dyslipidemia of high LDL and low HDL was 22.7%, while that of high TG and high LDL-C was 12%, and that of a combination of high TG and low HDL-C was 10.3% [16]. Among isolated dyslipidemias, high LDL-C was seen in 15.6%, low HDL-C in 11.6%, and high TG in 4% of the Western Indian population [16]. Another American study showed that the prevalence of combined high LDL-C and low HDL-C was 10%, while that of elevated LDL-C and TG was 12%, and that of elevated TG and low HDL-C was 16%. Accordingly, the prevalence of isolated dyslipidemia was 23% for high LDL-C, 13% for low HDL-C, and 10% for high TG, respectively. When our results were compared, the combined and isolated dyslipidemias were similar [13]. In contrast, the prevalence of combined dyslipidemia (high LDL-C and low HDL-C) in the European and Canadian populations was lower than in our study (1.6%) [17]. The variation in the pattern of dyslipidemia may be due to differences in the geographical area, genetic factors, socioeconomic factors, different sample sizes, and cut-off values for abnormal lipid parameters.

In the present study, a longer duration of diabetes was found to be associated with poor glycemic control. The finding is consistent with the Ghouse et al. study, which showed the long duration of diabetes to be associated with poor glycemic control (HR=1.33 95% CI = 0.84-2.11, p=0.001) [18]. However, The Fremantle Diabetes Study (FDS) of western Australia reported an inverse association between the long duration of diabetes and poor glycemic control (HR=0.56 95% CI = 0.32-0.96, p=0.03) [19].

High AIP was the most common abnormality among the atherogenic indices. The prevalence of high AIP in our study was similar to that in the Iranian population (88.7%) with type 2 diabetes [10]. On the contrary, the frequency of high AIP was low in China (60.5%) [20], and the Malaysian population (16.4%) [21]. Baral et al. reported a higher prevalence of elevated TC/HDL-C ratio (80%), TG/HDL-C ratio (78.4%), and non-HDL-C/ HDL-C ratio (80%) than our study. On the contrary, elevated LDL-C/HDL-C ratio (23.2%) was comparably lower than our study [22].

The LDL-C is used as the surrogate marker of CAD [23]. The American Diabetes Association (ADA) 2023, recommended the goals of LDL-C <100, LDL-C <70, LDL-C <55 mg/dl based on the CV risk [12]. Although all study participants received statins in this real-world scenario, LDL-C targets were not met according to ADA. This could be due to poor compliance or inability to follow therapeutic plans. Mithal et al. reported that the frequency of patients achieving LDL-C <100 mg/dl and LDL-C <70 mg/dl targets were 50.2% and 22.8%, respectively, which was higher than our study [6]. The HDL-C target was achieved in 69.5% of women and 52.9% of men, which is comparatively higher in women and similar to men in our study [6]. A study from the Vermont Diabetes Information System (VDIS) trial reported that the target achieved of LDL-C<70 mg/dl was 15.7% comparably higher than our study [24]. The analysis of Learnings with Experts to Advance Diabetic Dyslipidemia Management (LEADD) study showed the target of LDL-C <55 mg/dl was achieved in 3.8%, which was similar to our study [25]. The ADA has also designated a value of HbA1C <7% as a target for optimal blood glucose control. Further, the American Association of Clinical Endocrinology (AACE) recommended HbA1C<6.5% as a goal of glycemic control [26]. In our study, the goal of HbA1C <7% was achieved in approximately 1/10 of the total study subjects, while the target goal of HbA1C<6.5% was achieved in 2.9%. The Phase 1 of the Indian Council of Medical Research India Diabetes (ICMR-INDIAB) study conducted in three states and one Union Territory of the Indian type 2 diabetes population showed the prevalence of good glycemic control (HbA1C<7%) of 31.1% in urban and 30.8% in rural subjects, which was higher than our study [27]. Further, Memon et al. reported a trend that the complexity of CAD increases with age, elevated HbA1C, high TG, increased LDL-C, and low HDL-C [28].

The pattern of dyslipidemia (single, combined, and mixed dyslipidemia) and atherogenic indices (TC/HDL-C ratio, TG/HDL-C ratio, LDL-C/HDL-C ratio, non-HDL-C/HDL-C ratio, and AIP) were not associated with poor glycemic control in diabetic dyslipidemia patients. Kebede et al. reported that patients with poor glycemic control were 1.3 times more prone to have abnormal lipids in type 2 diabetes among the Ethiopian population [29]. In contrast, Khot et al. showed that poor glycemic control had no statistically significant association with abnormal lipid parameters in diabetic dyslipidemia in South Indian patients (p=0.79)[30].

This study provides real-world data on the pattern of dyslipidemia, atherogenic indices, and their association with glycemic control in tertiary care institutes. Lipid subfractions were measured directly rather than by calculation. Our studies have some limitations. Firstly, the study was limited by its cross-sectional design, which does not allow us to conclude any causal effect in our results. Secondly, in this study, the genetic factors, physical activity, and dietary intake patterns were not taken into account. In the future, prospective cohort studies can be done along with physical activities and dietary intake patterns to investigate the prevalence and predictors of diabetes dyslipidemia.

## Conclusions

Mixed dyslipidemia (high TG, high LDL-C, and low HDL-C) was the most common form in diabetic dyslipidemia patients. The most common pattern in atherogenic indices observed was elevated AIP. The duration of diabetes >10 years was associated with poor glycemic control. However, the pattern of dyslipidemia and atherogenic indices were not associated with poor glycemic control.
